# My Experience with Black-leg Vaccine1Read at the meeting of the Nebraska Veterinary Medical Association, February, 1899.

**Published:** 1899-04

**Authors:** M. V. Byers

**Affiliations:** Osceola, Neb.


### MY EXPERIENCE WITH BLACK-LEG VACCINE.[1]

By M. V. Byers, V.S.,
OSCEOLA, NEB.

My experience with black-leg vaccine has been somewhat limited. Perhaps some of my brother- or fellow-practitioners have had more experience in this line than myself. During the months of January and February, 1898, I vaccinated nearly 600 head, ranging in age from six months to two and one-half years.

During the early part of January, 1889, I was called out to examine some cattle which had been dying of some unknown disease. Upon arriving I found several dead animals, and upon post-mortem examination I found the characteristic symptoms of black-leg, which are too well known to the profession to give here. In order to be doubly sure of my diagnosis, I procured some of the diseased tissue and sent it to Dr. Jones, of Rising City, for micro-

[1] Read at the meeting of the Nebraska Veterinary Medical Association, February, 1899.

scopic examination, and received an answer confirming my diagnosis. This herd consisted of about 150 head. I advised vaccination. The owner had never heard of such a thing, so it was impossible to convince him. He said that if it was nothing worse than black-leg he could cure them, so I said, "Go ahead." I do not know what his treatment consisted of, but I do know that he lost about twenty-two head of nice steers. They kept on dying all winter.

The next herd was about two miles from the first, and I will say the owner was a more sensible man to deal with. He had lost several head. We vaccinated 97 head with first lymph (I will say right here that I have used the double vaccine altogether, and think it best); 3 died between the first and second vaccinations; six or eight weeks after the second, 2 died; none from that time up to this.

In the second herd of 57 heifers, 2 died before vaccination; none during the intervals, and none after that. In the next herd of 354 head, 12 died prior to vaccination; none during the interval; 1 after complete vaccination.

I also vaccinated several smaller herds, with no deaths after vaccination. I have always used the Pasteur vaccine, but there may be others just as good. It will be seen from the above that there were only three deaths after complete vaccination out of nearly 600 head, and the disease actually existed in all of those herds pior to vaccination.

Some would ask how many can be vaccinated in a day. I will say that that depends upon the surroundings and the help you have. I vaccinated 354 in less than ten hours, and others can do equally as well if they have the proper assistance.

There are three modes of vaccination: the ear, shoulder, and tail. I prefer the latter for several reasons.

I will further state that it is generally believed that cattle will not contract the disease after three years of age. I will relate an instance which occurred in one of the herds. There were about ninety head of cows—very wild Western cows. In separating the young stuff to be driven into another corral to be vaccinated, these cows stampeded and broke through a six-wire fence, and right by this fence lay a carcass of one of the diseased animals; those cows were all more or less scratched by the wire; those cows were sold about two days later, and in about two weeks six of them died, as I am told, with all the characteristic symptoms of black-leg. This proves the theory of its being an infectious disease of wounds.

I might further be asked if it is safe to vaccinate pregnant heifers. I will say that I think it is, as I vaccinated over sixty, and not a single abortion followed, to my knowledge.

I will close by saying that I heartily indorse black-leg vaccine where the disease actually exists.

